# Effects of disinfection and sterilization on the dimensional changes and mechanical properties of 3D printed surgical guides for implant therapy – pilot study

**DOI:** 10.1186/s12903-020-1005-0

**Published:** 2020-01-23

**Authors:** Gréta Török, Péter Gombocz, Eszter Bognár, Péter Nagy, Elek Dinya, Barbara Kispélyi, Péter Hermann

**Affiliations:** 1 Department of Prosthodontics, Faculty of Dentistry, Semmelweis University, Szentkirályi Street 47, Budapest, H-1088 Hungary; 2 Department of Materials Science and Engineering, Faculty of Mechanical Engineering, Budapest University of Technology and Economics, Bertalan Lajos Street 7, Budapest, H-1111 Hungary; 3 Budapest and MTA–BME Research Group for Composite Science and Technology, Műegyetem rkp. 3, Budapest, H-1111 Hungary; 4 Institute of Digital Health Sciences, Semmelweis University, Ferenc Square 15., Budapest, H-1094 Hungary

**Keywords:** 3D printing, Dental implantology, Drill template, Sterilization, Disinfection

## Abstract

**Background:**

The purpose of this research was to investigate the effects of disinfection and three different sterilization methods on the dimensional changes and mechanical properties of three-dimensional (3D) printed surgical guide for implant therapy. The objective was to assess the effects of sterilization procedures in 3D printed drill guide templates with destructive and non-destructive material testing.

**Methods:**

Fifteen identical drill guide templates were produced using a 3D printer. The surgical guides were classified into five groups: three controls, three disinfected (4% Gigasept®, 60 min), three plasma sterilized, three autoclave sterilized (+ 1 bar, 121 °C, 20 min), and three autoclave sterilized (+ 2 bar, 134 °C, 10 min).

The templates were digitalized with a Steinbichler SCAN ST 3D scanner. Length was measured under an SZX16 stereomicroscope. A scanning electron microscope was used to study the surface morphology of the drill templates. The hardness, and flexural and compressive strength were measured to assess any changes in the physical characteristics of the material caused by sterilization. The drill guide templates were also examined with a Dage XiDAT 6600 X-ray. During the X-ray examinations, the following parameters were used: 100 kV voltage, 128 AVG averaging, 0.8 W power. One-way analysis of variance (ANOVA) was used to detect the difference between groups.

**Results:**

Evaluation of the hardness measurements of the various specimens shows that the hardness of the material was not changed by the plasma sterilization (*p* = 0.0680), steam sterilization on 121 °C (*p* = 0.6033) or disinfection process (*p* = 0.1399). The statistical analysis revealed significant difference in hardness strength of the autoclave sterilized (134 °C) specimens (*p* = 0.0002). There was no significant difference between the goups regarding the scanning electron microscopic and stereomicroscopic examinations. There was no significant difference regarding the X-ray visibility of the templates to the effect of the disinfection (*p* = 0.7844), plasma sterilization (*p* = 0.4091) and steam sterilization on 121 °C (*p* = 0.9277) and steam sterilization on 131 °C (*p* = 0.093). The effect of the sterilization was the same in case of both flexural and compressive strength of the material.

**Conclusions:**

Our findings indicate that plasma sterilization and steam sterilization at 121 °C were both suitable for sterilizing the tested 3D printed surgical guides.

## Background

Modern 3D imaging technologies and software can be great assistance in the pre-operative planning of dental implant surgery [1, 2]. The virtually planned implant position is transferred to the surgical area by using an implant drill guide template, and resulting greater control of the implant procedure [3, 4]. Implant surgery is an invasive procedure, during which the surgical templates come in contact with blood, injured mucous membranes, and bone. If the drill template is not sterilized properly, microorganisms can easily enter to the surgical wound and negatively affect the success of the surgery and the lifespan of the implant. Therefore, like all other instruments used in the implant surgery, drill templates should be also sterilized to avoid infection [5].

Sterilization is the complete elimination or destruction of all forms of microbial life and it is accomplished in healthcare facilities by either physical or chemical processes. Steam under pressure, dry heat, ethylene oxide (ETO) gas, hydrogen peroxide gas plasma, and liquid chemicals are the principal sterilizing agents used in healthcare facilities. Disinfection describes a process that eliminates many or all-pathogenic microorganisms on inanimate objects with the exception of bacterial spores. Unlike sterilization, disinfection is not sporicidal. Disinfection is usually accomplished by the use of liquid chemicals or wet pasteurization in healthcare settings. Disinfection differs from sterilization by its lack of sporicidal property. A few disinfectants can kill spores with prolonged exposure times (3–12 h) and are called chemical sterilants [6]. The International Organisation for Standardization (ISO) defines the sterile in the ISO/TS 11139:2018 document, as sterile is from viable microorganisms [7]. The European Standard (EN 556–1:2001) specifies the requirements for a terminally sterilized medical device to be designated sterile. According to this standard sterile is defined as the theoretical probability of there being a viable micro-organism present on/in the device shall be equal to or less than 1 × 10^–6^ [8]. The Official Journal of the European Union published The European Medical Device Regulations in 2017. The aim of this regulation is to set a high standards of quality and safety for all medical devices and ensure the protection of health for patients and users whilst supporting innovation [9]. In addition, the International Organisation for Standarization is regularly updating standards for processing of health care products to provide information for the medical device manufacturers. The latest standard in this field was published in 2017. ISO 17664:2017 specifies requirements for the information to be provided by the medical device manufacturer for the processing of a medical device that requires cleaning followed by disinfection and/or sterilization to ensure that the device is safe and effective for its intended use. The ISO 17664:2017 standard is to be applied to medical devices that are used for invasive or direct/indirect patient contact [10]. These regulations and international standards are known and followed for every manufacturers and users [7–10].

Suppliers recommend disinfecting or sterilizing the drill templates before surgery. According to manufacturers’ recommendations ethanol solution and non-chemical products are preferred if disinfection methods are used to. It should also be considered that drill templates have porous surfaces that are difficult to disinfect [5]. Therefore sterilizing drill templates before surgery should be considered. The commonly used biocompatible 3D printing materials are MED 610 (Stratasys, Eden Prairie, MN, USA) and Dental SG resin (Formlabs, Somerville, MA, USA). However, 3D printed drill guide templates composed of materials such as polymer MED 610 (Stratasys, Eden Prairie, MN, USA) have an upper temperature limit of form stability of 45–50 °C according to the material data sheet (ASTM D648–06) [11]. Suppliers of MED 610 recommendation is using steam sterilizator for 4 minutes at 132 °C with fractioned pre-vacuum or gamma sterilization using a dose of 25–50 kGy. They note that autoclave may cause deformations and changes to be flexural strength. It is noted that gamma sterilization may result color changes. Dental SG 3D printed drill guide can be sterilized with autoclave at 121 °C for 15 min or at 134 °C for 6 min, and at 138 °C for 3 min. In case of Dental SG resin steam sterilization may result the change of color. The application guide mentioned if disinfection methods is preferred ethanol solution may be recommended [12]. The SprintRay (Los Angeles, CA, USA) manufacturer’s recommendtaions for preparing the 3D printed surgical guides are also disinfection with ethanol solution or steam sterilization before the implant surgery [13]. If the drill guide template is deformed and its physical features are modified during the sterilization procedure, it will affect the accuracy of the implantation. That is why important to investigate the effects of the different sterilization methods especially on 3D printing drill guide templates.

The purpose of the research was to investigate the effects of disinfection and different sterilization methods on the dimensional changes and mechanical properties of 3D printed surgical guide for implant therapy. Our study investigated four options for sterilizing 3D printed surgical guides, with the aim of finding a method that can sterilize the templates without damaging and deforming them. The research included destructive and non-destructive material tetsing and geometric analysis of the effects of disinfection, plasma sterilization, and autoclave-based steam sterilization on surgical guides. The null hypothesis was that low-temperature sterilization may be suitable for preparing 3D printed surgical guides for implant surgery without damage. Futhermore, high temperature steam sterilization may cause deformation and damages in 3D printed guides. The heat sensitive polymer can probably not be sterilized in a high-temperature and high-pressure autoclave without suffering any deformation.

## Methods

### Drill templates

Fifteen dental implant drill guide templates of identical size and shape were manufactured for the purpose of the tests. The drill templates were produced with an Objet Eden 350 V type printer (Stratasys) and PolyJet technology (Stratasys, Eden Prairie, MN, USA). The resolution was 16 μm. The 3D-printed surgical templates were made of Objet MED 610 material (Stratasys). The 3D printed sugical guides are provided by Merfol ltd. (Budapest, Hungary). We divided the specimens into five groups. Three specimens served as controls (marked A, B, and C). These were neither disinfected nor sterilized. Three specimens (D, E, and F) were disinfected, they were soaked in a 4% disinfectant solution (Gigasept® Instru AF; Schülke & Mayer Gmbh, Norderstedt, Germany) for 60 min. Specimens J, K, and L were sterilized with a plasma sterilizer (Sterrad 100NX; ASP, Irvine, CA, USA) at 46 ± 4 °C for 50 min. Six specimens were sterilized by autoclaving using a Sanamij SAR 6.6.9-2 V autoclave (Gemini BV, Apeldoorn, The Netherlands) in compliance with the specifications. Three of these specimens (G, H, and I) were sterilized at a pressure of + 1 bar and a temperature of 121 °C for 20 min; and three specimens (M, N, and O) were exposed to + 2 bar pressure and 134 °C temperature for 10 min.

### 3D scanner examination

**Fig. 1:**
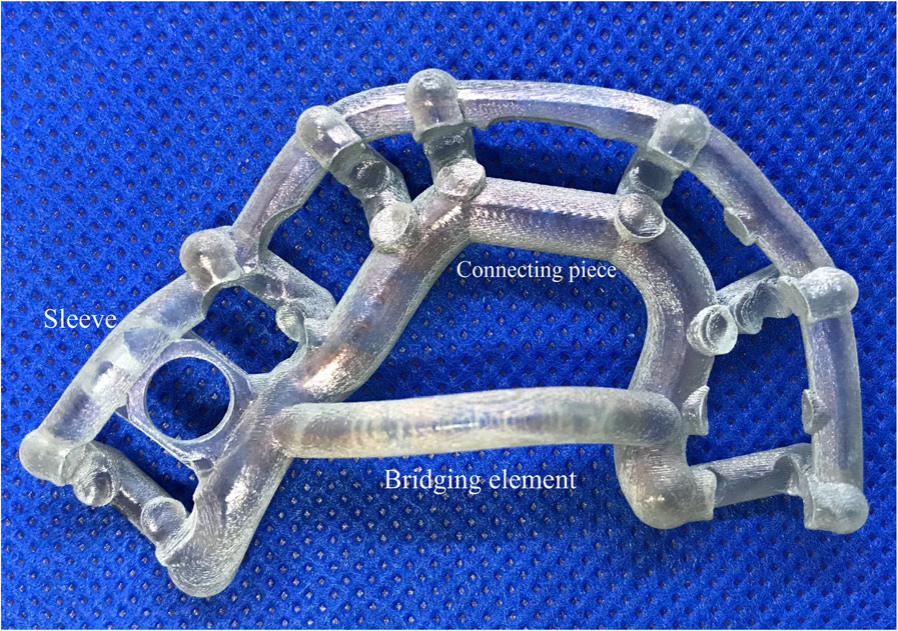
The figure shows the parts of 3D printed surgical guides were signed where the measurements were carried out

The 3D-printed drill templates were digitally scanned by using a Steinbichler Scan ST dental scanner (Steinbichler, Neubeuern, Germany), which allowed 3D models with 18-megapixel resolution to be produced. The records were saved in stereolithography (STL) file format. Using MeshLab software (GNU General Public License Version 2.0), we performed sub-millimeter measurements at the locations on the templates expected to be the most exposed to damage. A critical region of the drill template is the sleeve that guides the surgical drill when creating the bone cavity for the implant during the implantation procedure. Therefore, it is essential to record the spatial data of the holes because any deformation in this area may change the direction of the implantation, thus affecting the accuracy of the procedure. The region that bridges the palate and connects the distal sections serves only as reinforcement for the template; however, owing to its dimensions, any deformation that occurs can be best measured here. Thus, we also measured the span and diameter of this section. We also recorded reference data at the connecting section at the frontal region of the template. Figure 1 shows the locations of the 3D printed specimen where the measurements were specified. After the templates were sterilized, we re-scanned the specimens and repeated the measurements and calculations. Three of the available surgery drill templates (J, K, and L) were plasma sterilized. The parameters were configured the same way on the 3D images of these samples as above. The data measured on the plasma sterilized specimens were compared with those of the control specimens A, B, and C. All measurements were performed ten times for each template.

### Stereomicroscopic examination

**Fig. 2:**
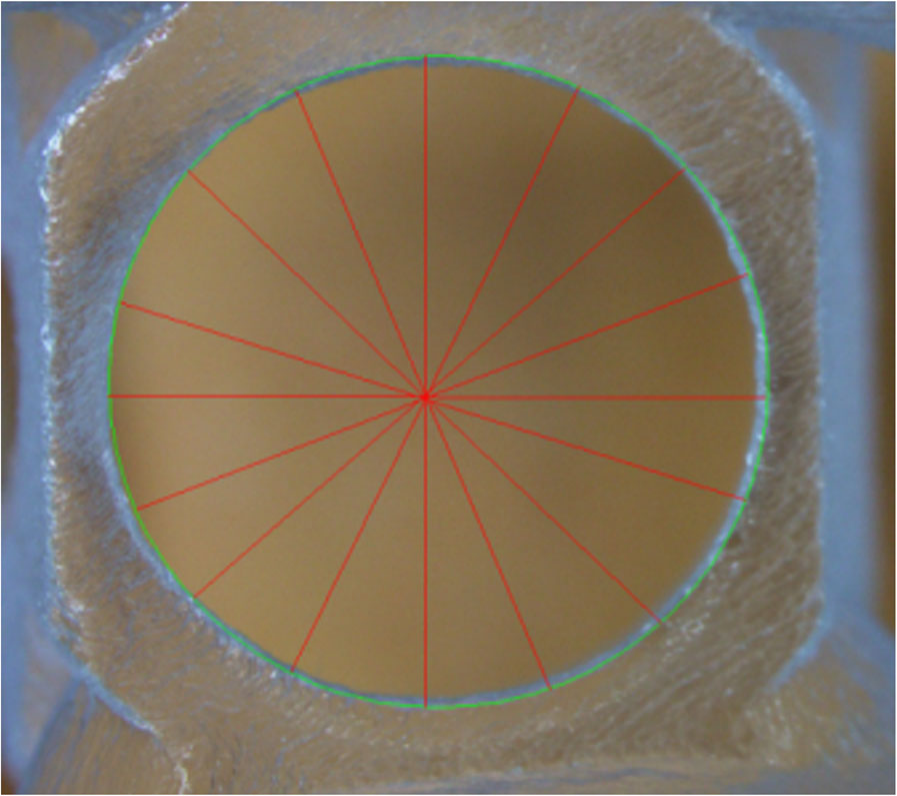
The figure shows how the stereomicroscopy examination was done. The dimensions of the hole was recorded. The stereomicroscopic measurements were performed on the specimens before and after disinfection or sterilization. For each drill guide template measurements were performed eigth times

Surface and geometric features of the implant drill guide templates were studied with an Olympus SZX 16 stereomicroscope (Olympus, Tokyo, Japan). We also recorded the dimensions of the hole and the bridging element stereomicroscopically. Dimensional data were obtained using the JMicroVision 1.2.7 program (JMicrovision, Geneva, Switzerland). The stereomicroscopic measurements were performed on the specimens before and after disinfection or sterilization. For each drill guide template measurements were performed eigth times (Fig. 2).

### X-ray microscopic examination

We examined the implant drill guide templates with a Dage XiDAT 6600 X-ray microscope (1st Place Machinery, Middleton, MA, USA), which provided a comprehensive image of the templates. During the X-ray examination, the following parameters were used: 100 kV voltage, 128 AVG averaging, and 0.8 W power. Six images were taken for every specimen. For each settings, one image with and one without a contrast agent was taken. A method developed by the Department of Materials Science and Technology of Budapest University of Technology and Economics makes it possible to objectively evaluate X-ray visibility of the specimens [14]. The extent of visibility, expressed as a percentage, can be easily compared. The visibility study was performed on the same three parts of every specimen. The X-ray visibility was determined from the images without a contrast agent. The program expresses X-ray visibility of the specimens as a percentage, relative to the background. The values measured at different locations on the sample are of the same order.

### Scanning electron microscopic examination

The implant drill guide templates were studied with a Philips XL 30 scanning electron microscope (Philips Electron Optics, Cincinnati, Ohio, USA). The surface analysis was carried out by an energy dispersive X-ray detector installed on the scanning electron microscope. The photographs were taken in the secondary electron (SE) mode with 15 kV accelerating voltage. Images were taken of every template before and after disinfection or sterilization. The studied specimens were made of a non-conductive polymer; therefore, the templates had to be plated with several nanometers thickness of gold before scanning. The gold was vaporized in the vacuum at a current of 45 mA for 25 min. The conductive layer was applied using a BAL TEK SCD 005 (BalTec Maschinenbau AG, Pfäffikon ZH, Switzerland) device. Scanning electron microscopes have a high resolution, and were therefore appropriate for studying the surface morphology and structure of the implant drill templates in detail. The microscope helps to identify structural difference, material defects, cracks, and contaminations.

### Hardness measurement

To measure hardness, metallographic sections were prepared from the drill templates. Using a diamond-disk cutter (Buehler IsoMet 1000; Buehler, Lake Bluff, IL, USA), small samples were cut from three different areas of the drill templates to compare the hardness at different locations. Three small samples cut out of each template were embedded in a single disk. The samples were polished with course, then finer, silicone carbide sandpapers (P600, P1200, P2500). They were then polished with a paste containing 1–3-μm aluminum oxide grains (Topol 1, Buehler, Lake Bluff, IL, USA). Vickers hardness testing has been performed on various polymer samples in previous studies [15, 16]. We measured the Vickers hardness (HV) test with a Buehler Micro Vickers hardness tester (Buehler, Lake Bluff, IL, USA), consisting of a square-based diamond pyramid with a vertex angle of 136°. To improve the visibility of the diamond pyramid, a nanometer-thick gold layer was evaporated onto the surface of the metallographic sections with the BAL TEK SCD 005 device (45 mA, 25 s). The diamond pyramid was pressed into the samples with a force of 50 g for 5 s. Five measurements were taken for each sample, and the two diagonals of the impression and the micro-hardness values were measured and averaged.

### Flexural strength measurement

A flexural stress test was used to determine the resistance of the material to flexural. The rigidity of the samples was evaluated by a three-point flexural test using an Instron 5965 twin-column device (Instron, Norwood, MA, USA). Each sample was placed accurately on two rounded-tip edge-like supports (cross-section of 2 mm), and then a third similarly shaped edge was applied centrally between the two supports, exerting a continuous load of 5 mm/min until the sample broke. The samples from the bridging element (number 1) were much longer than the number 2 samples, which had been cut out of the central section of the outer arch.

### Compressive strength measurement

The compressive strength of the polymer implant drill guide templates was determined using an Instron 5965 dual column testing system (Instron, Norwood, MA, USA). The sample was placed vertically between the clamping heads, and a horizontal metal plate exerted a continuous compressive load of 5 mm/min on the tightly clamped sample until the sample broke.

### Statistical analysis

Deviation measurement data were described using descriptive statistics (means±standard deviations) for each sterilization method. The parametric or the non-parametric equivalent (Kruskal-Wallis test) one-way analysis of variance (ANOVA) was used to detect the difference between groups. Since it is a non-parametric method, the Kruskal–Wallis test does not assume a normal distribution of the residuals. Tukey’s HSD and Mann–Whitney Upost hoc tests were performed after a statistically significant one-way ANOVA result to confirm where the difference occurred betweengroups. At Mann–Whitney U test we have used Bonferroni correction. Analyses were performed with the computer software IBM SPSS Statistics (Version 25). Significance criterion was set at α = 0.05.

## Results

### 3D scanner

**Fig. 3:**
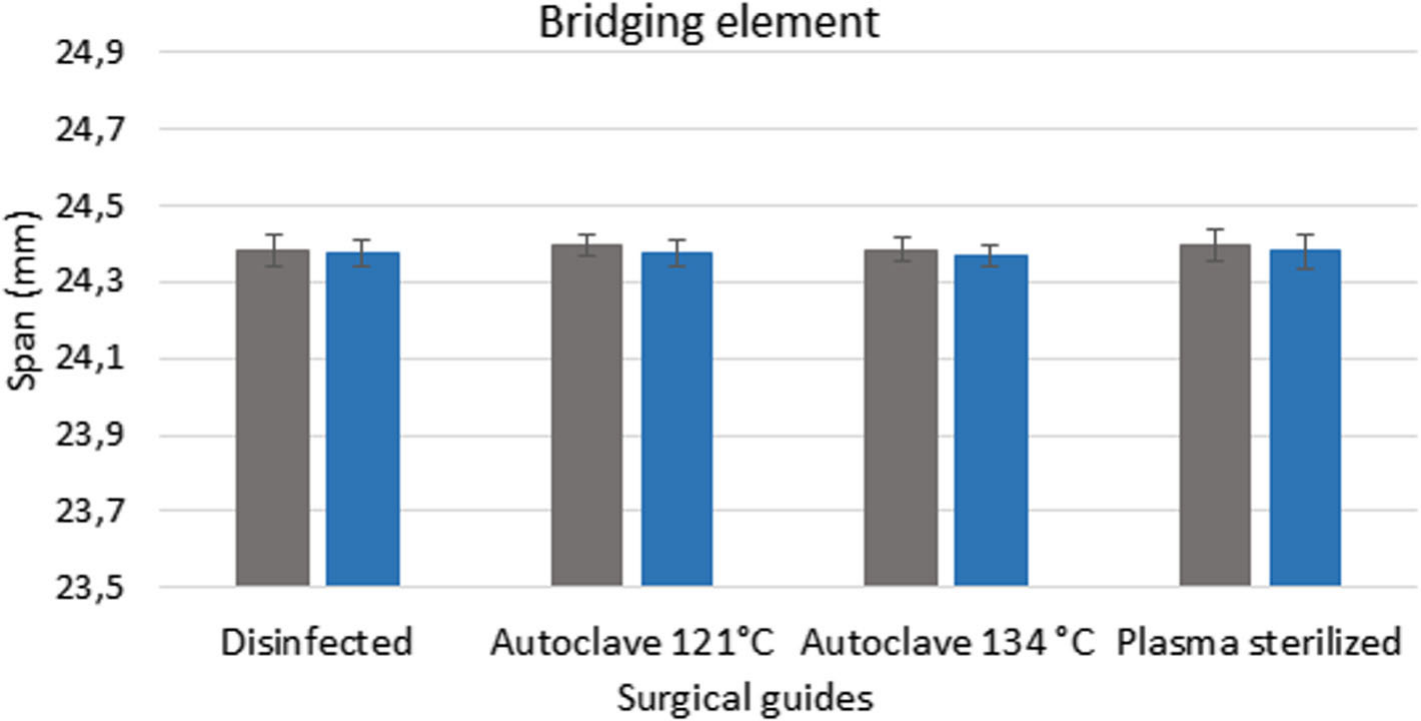
Results of 3D scanner examinations. The diagram shows the span (mm) of the bridging element before and after disinfection and sterilization. The reference data (grey column) were obtained before sterilization. The data, measured after sterilization and disinfection, are showed in the blue columns

**Fig. 4:**
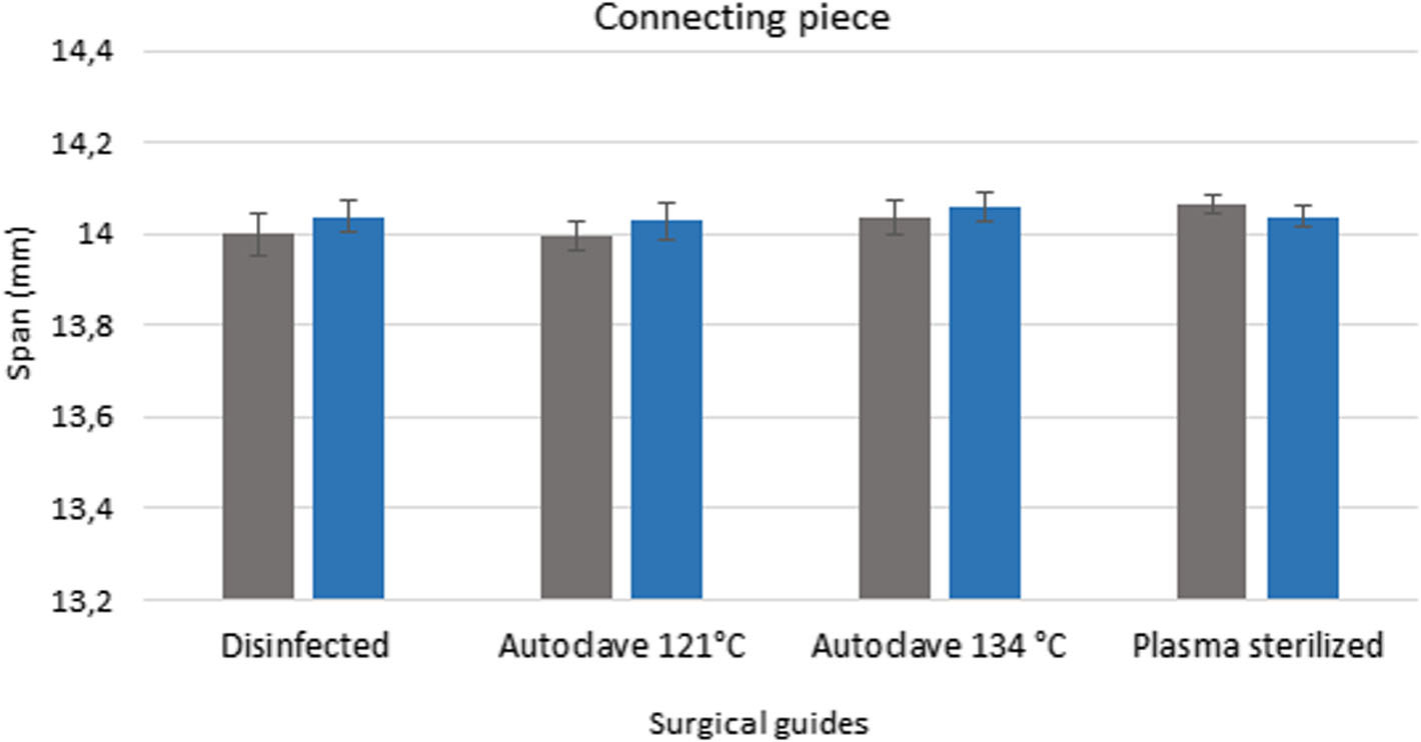
Results of 3D scanner examinations. The diagram show the span (mm) of the connecting piece of the surgical guides before and after disinfection and sterilization. The diagram shows the data measured after sterilization (blue columns) and the reference data (gray columns)

**Fig. 5:**
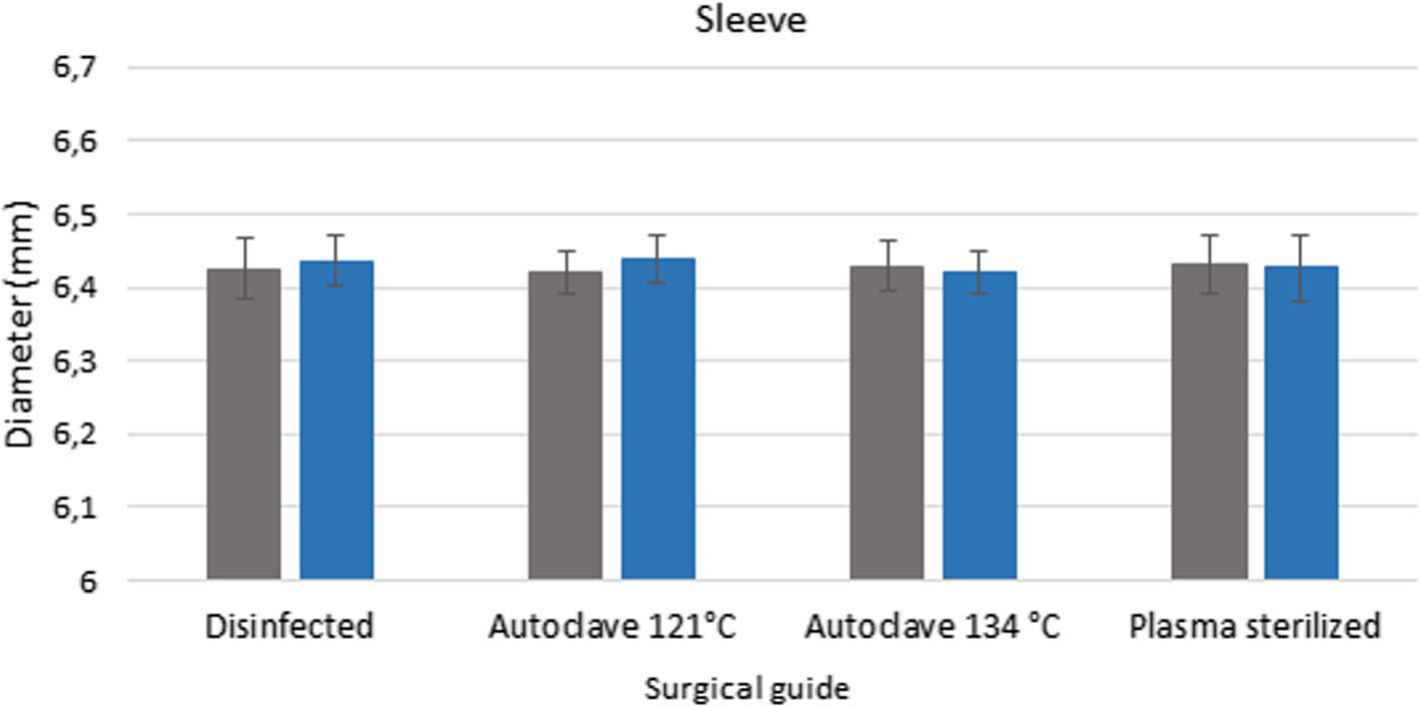
Results of 3D scanner examinations. Diameter (mm) of the sleeve of disinfected and sterilized specimens compared with the dimensions before the sterilization and disinfection procedures. The diagram shows the data measured after sterilization (blue columns) and the reference data (gray columns)

Figures 3, 4 and 5 show the data measured after sterilization (blue columns) and the reference data (gray columns). The reference data (grey column) were obtained before sterilization in the groups of disinfected guides and sterilized templated with autoclave. In the group of plasma sterilized templates the reference data were obtained from the control group. Each columns of diagrams show the mean and standard deviation of ten measurements. None of the tests yielded significant defects, indicating that the templates remained free of deformations during sterilization. ANOVA was used to prove the 3D printed drill guide template were identical. The results indicate that there is no significant difference between the templates before and after disinfection and sterilization (*p* > 0.05).

### Stereomicroscopy

**Fig. 6:**
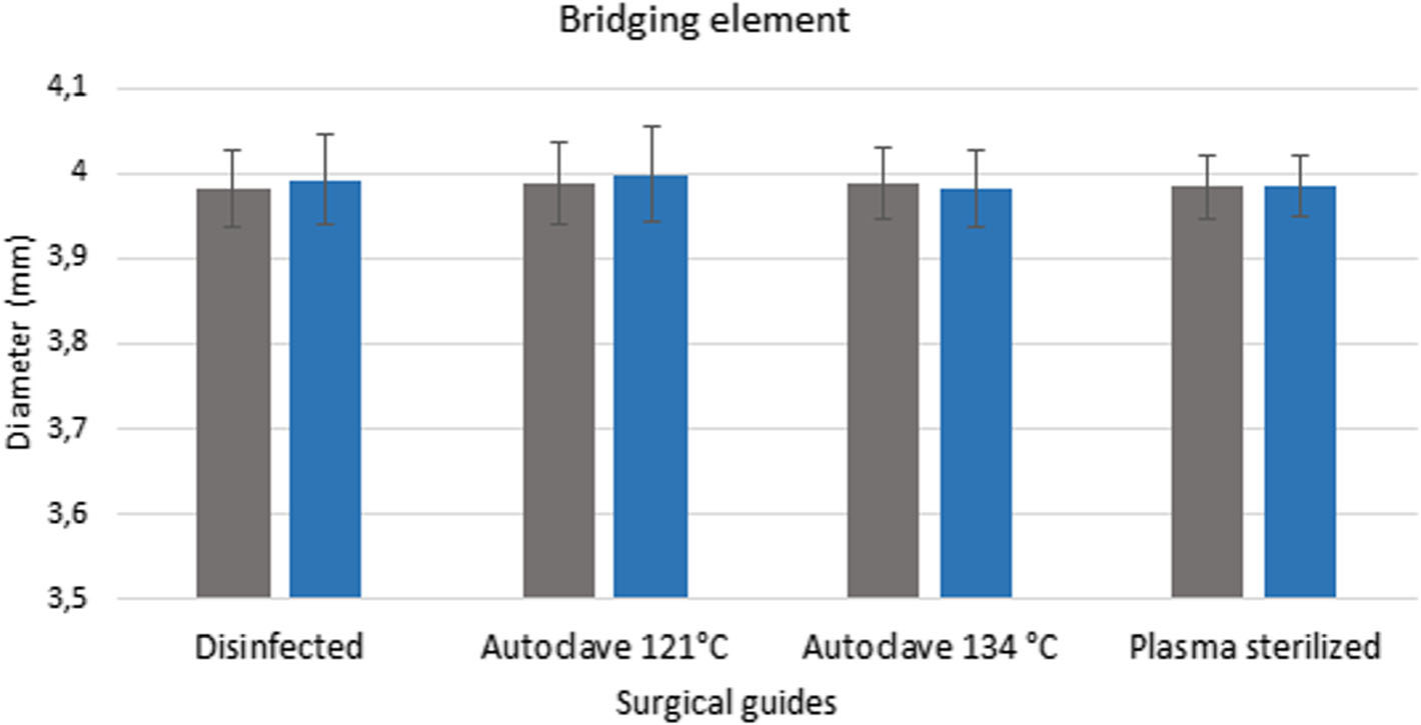
Results of stereomicroscopic examinations. Span (mm) of the bridging element of disinfected and sterilized specimens compared with the dimensions before the sterilization ans disinfection treatments. The diagram shows the data measured after sterilization (blue columns) and the reference data (gray columns)

**Fig. 7:**
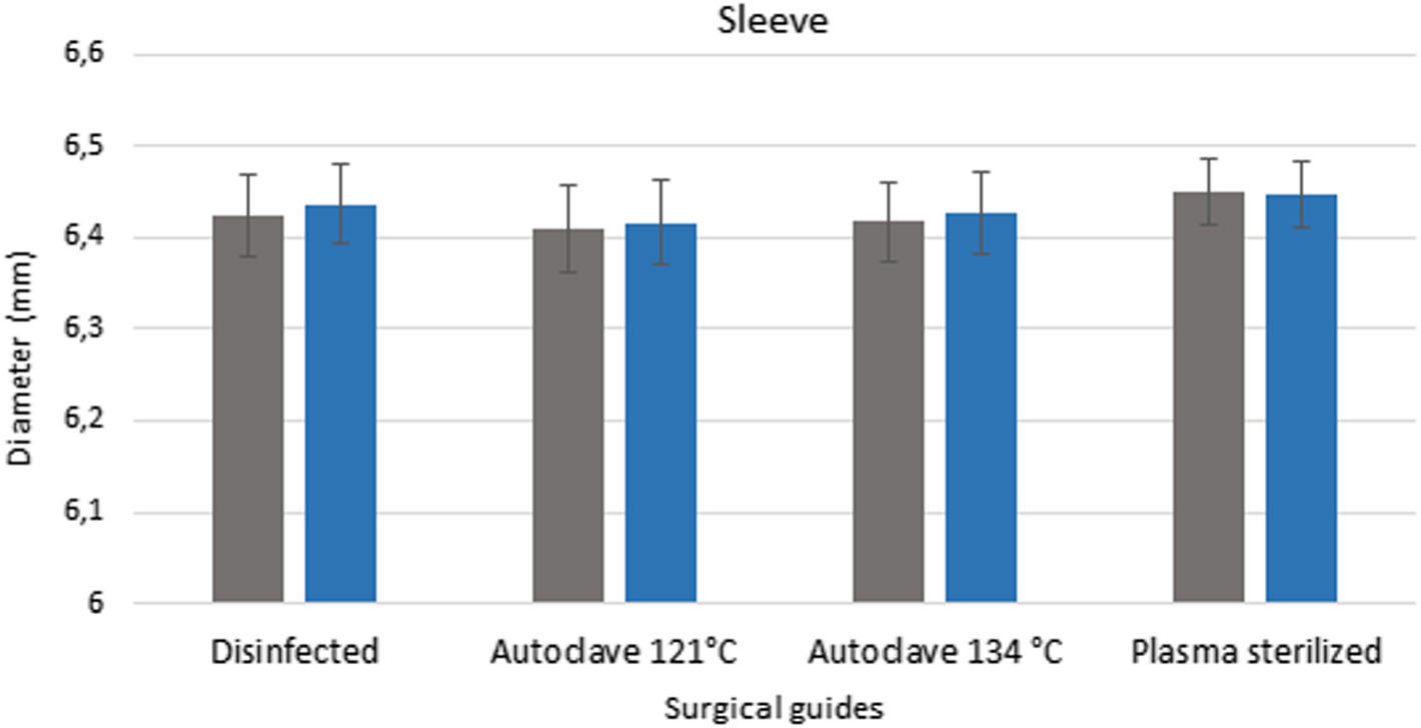
Results of stereomicroscopic examinations. Diameter of the sleeve of disinfected and sterilized specimens compared with the dimensions before the sterilization and disinfection procedures. The diagram shows the data measured after sterilization (blue columns) and the reference data (gray columns)

Pre-treatment measurement data form the basis for determining the extent of any deformation caused by sterilization. The values measured after disinfection and sterilization, along with the reference data, are displayed in a bar graph (Figs. 6 and 7). Figures show the data measured after sterilization (blue columns) and the reference data (gray columns). Each columns of diagrams show the mean and standard deviation of the measurements. These results indicate that neither disinfection nor plasma nor steam sterilization resulted in any significant changes or deformation. The results indicate that there is no significant difference between the dimension of the guides before and after disinfection and sterilization (p > 0.05).

### X-ray microscopy

X-ray microscopy showed no dissolutions or inclusions in the material. Evaluation of the visibility parameters obtained by the measurements shows that X-ray visibility of the samples was extremely low. X-ray visibility of the implant drill guide templates ranged from 1.5 to 3.5%, which is a realistic value in polymer specimens. Before and after sterilization and disinfection of the 3D-printed drill guide templates were examined with X-ray microscopy. The visibility was measured on the same three parts of every template. Table 1 shows the mean and standard deviation of the measured X-ray visibility of the controls, the disinfected samples, the plasma sterilized templates and the steam sterilized drill guide templates. Comparison of the X-ray visibility of the control specimens shows that there is no significant difference between the plasma sterilized templates (*p* = 0.409). There is also no significant difference regarding the X-ray visibility of the templates to the effect of the disinfection (*p* = 0.784) and steam sterilization on 121 °C (*p* = 0.927) and sterilization with autoclave on 131 °C (*p* = 0.093).

**Table 1:** The mean, standard deviation (Std. Dev.) and *p* value of the measured X-ray visibility

	Mean	Std. Dev.	p
Control	2.0522	0.4365	–
Disinfected	2.11	0.4444	0.784
Plasma sterilized	2.2866	0.6653	0.409
Autoclave 121 °C	2.0244	0.6529	0.927
Autoclave 134 °C	2.0477	0.568	0.093

### Scanning electron microscopy

**Fig. 8:**
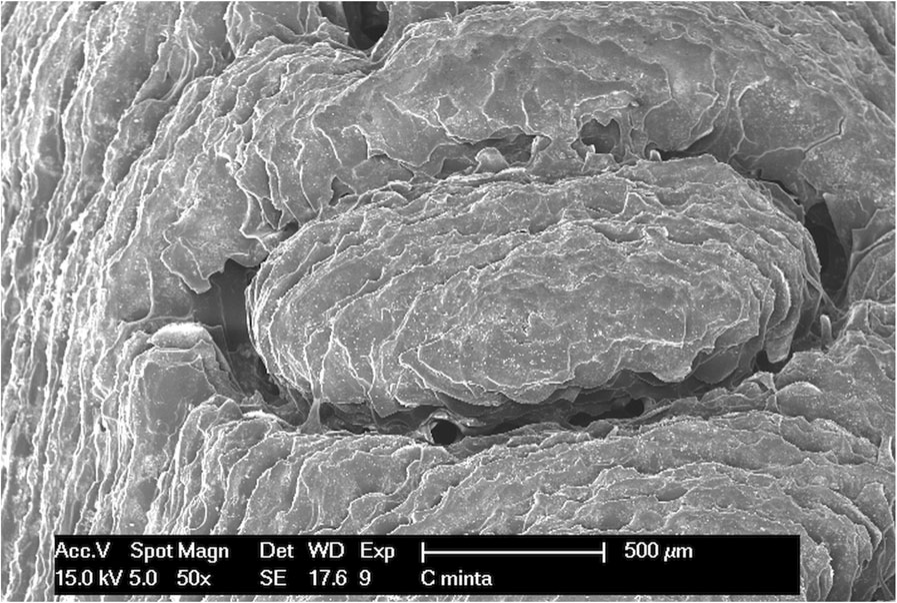
Scanning electron microscope (SEM) image of control specimen

**Fig. 9:**
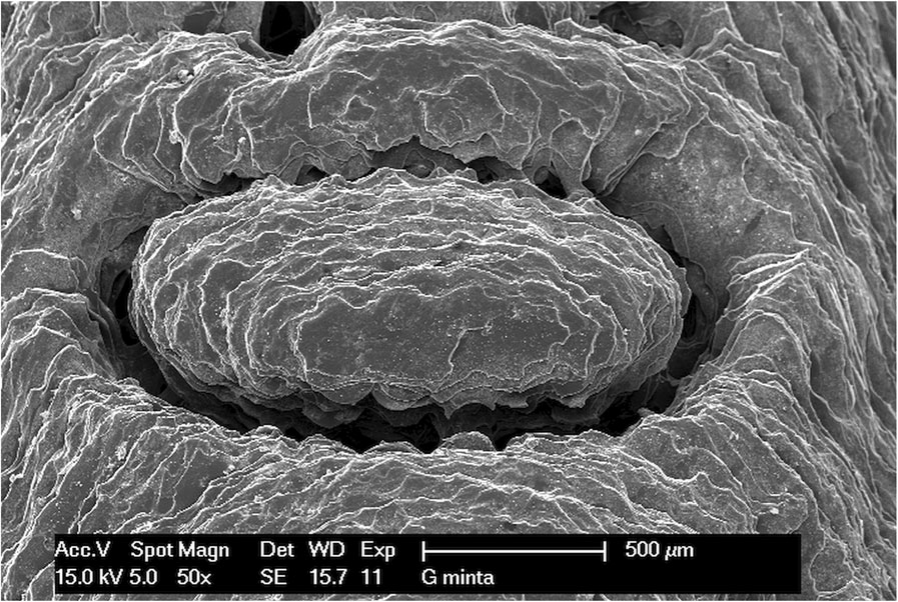
SEM image of the autoclave-sterilized specimen (121 °C)

**Fig. 10:**
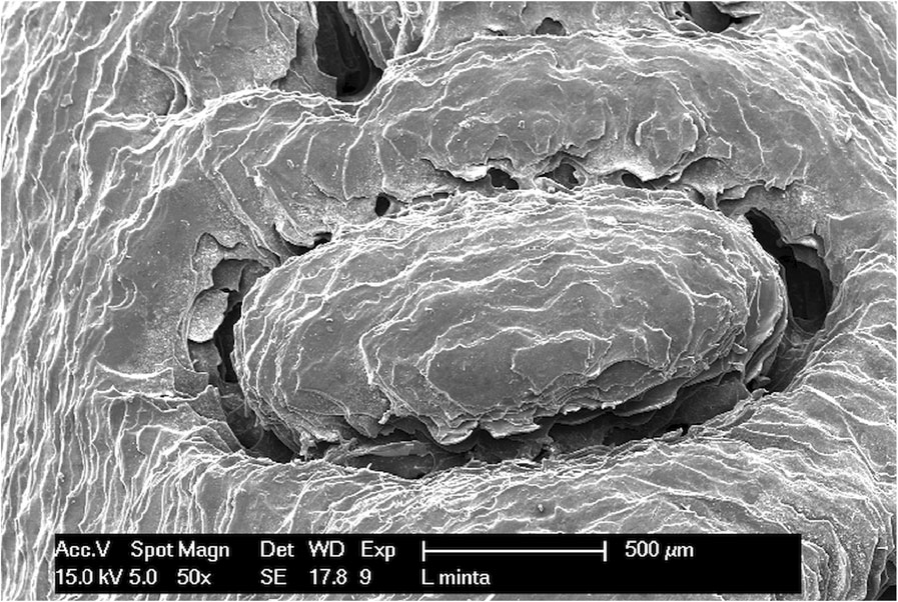
SEM image of plasma sterilized specimen

**Fig. 11:**
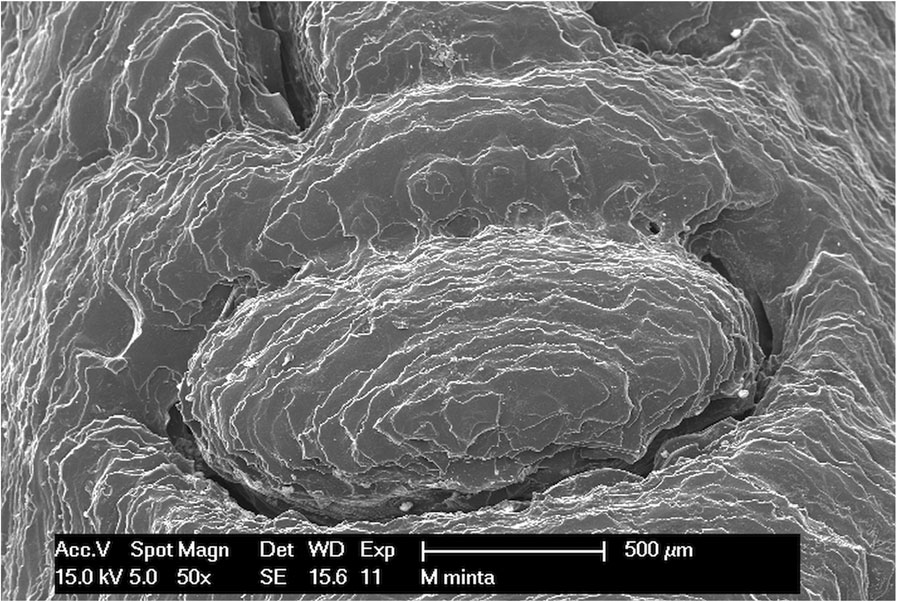
SEM image of the autoclave-sterilized specimen (134 °C)

Figures show one letter of the patient’s name, pressed into the bridging element, at 50× magnification. The following images (Figs. 8, 9, 10 and 11) show scanning electron microscopy images of the bridging element after sterilization. These images show the characteristic layered structure of the specimens and the groove around the letters. Scanning electron microscopy images of specimens soaked in disinfectant solution shows no morphologic changes. The plasma-sterilized specimens also show no surface changes. Comparison of images taken after autoclave sterilization with the baseline control images indicates that the specimens did not suffer any damage or morphologic changes. Despite the high-temperature treatment, the heat-sensitive polymer did not melt, and the layered structure of the specimens is still clearly visible.

### Hardness measurement

Three small samples cut out of each drill template were embedded in a single disk. Five measurements were taken for each sample. The Table 2 shows the mean and standard deviation of the measured data in Vickers. In summary, the average measured hardness (HV = 10.1 ± 1.10 Vickers) is relatively low. The 3D printed surgical guide were made of polymer, which is expected to record a low hardness score. Difference in hardness values within 5 Vickers are permitted in polymer specimens and caused by the natural flexibility of the material. Therefore, no real decrease or increase in the material’s hardness took place. Evaluation of the hardness measurements of the various specimens shows that the hardness of the material was not changed by the plasma sterilization (*p* = 0.068), steam sterilization on 121 °C (*p* = 0.603) or disinfection process (*p* = 0.139). The analysis revealed a statistically significant difference in hardness between the control and on 134 °C autoclaved specimens (*p* = 0.0002).

**Table 2:** The results of the hardness measurements

	Mean	Std. Dev.	*p*
Control	10.3333	0.9484	–
Disinfected	9.88	0.6603	0.139
Plasma sterilized	9.76	0.6843	0.068
Autoclave 121 °C	10.1666	0.7808	0.603
Autoclave 134 °C	11.5533	0.4549	0.0002

Legend: Mean and standard deviation (std. dev.) of tested samples in Vickers. Three small samples were cut out of each drill template. Five measurements were taken for each sample.

### Flexural strength test

**Fig. 12:**
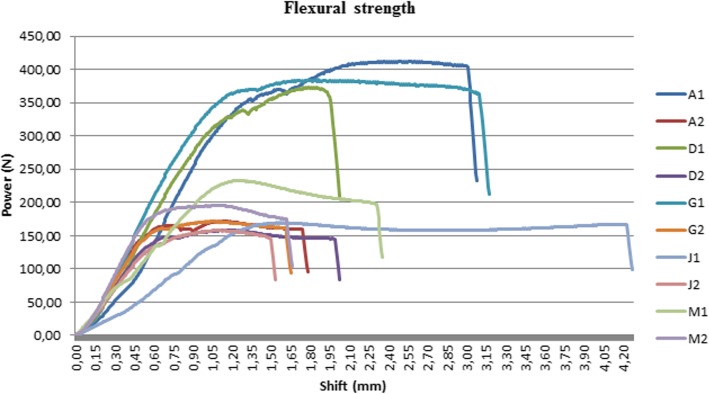
Flexural strength test results

Figure 12 shows that the loading force increased gradually, then the curve suddenly breaks at the point at which the samples snapped. The maximum flexural torque occurred at this point. No. (number) 1 samples had been cut out of the bridging element of the drill template and showed a higher resistance than no. 2 samples, which had been cut out of the central section of the outer arch. This difference in the flexural strength may have been caused by the difference in the lengths of the samples: no. 2 samples were smaller, and thus snapped at a lower force. The samples from the bridging element (no. 1) were longer than the no. 2 samples; therefore, higher flexural forces were present in these samples. The results of the flexural strength test are shown in Table 3. In case of bridging element (no. 1), the maximal flexural forces were very volatile, but in case of central section of the outer arch (no. 2) they were homogeneous; the maximal flexural forces were not depended on the type of sterilisation. In case of bridging element’s samples, the maximal flexural forces were decreased by sterilisation in the smallest case only 7% (in case of Autoclave 121 °C) and at most 59% in case of plasmas sterilisation. By central section of the outer arch, the Autoclave 134 °C increased the maximal flexural force (by 14%), the other types decreased this force in the worst case only by 8%. Compared to the maximal flexural forces in case of bridging element and central section of the outer arch, the bridging element’s values were higher than central section of the outer arch for all types of sterilization, which could be caused by the sample sizes (samples from the bridging element were much longer). Forces were homogeneous by plasma sterilisation and low heterogeneous by Autoclave 134 °C, the others were very volatile. There was any correlation between the bridging element’s forces and central section of the outer arch’s forces (R2 = 0.017).

**Table 3:** Measured flexural forces before and after sterilization

Sterilization type	no. 1		no. 2		Comparison	
	Strength (N)	Decreasing (%)	Strength (N)	Decreasing (%)	CV	Difference (%)
Control	412.9	0	171.5	0	0.58	141
Disinfection	373.5	10	158.8	7	0.57	135
Plasma sterilized	169.7	59	158.3	8	0.05	7
Autoclave 121 °C	384.3	7	171.3	0	0.54	124
Autoclave 134 °C	232.8	44	195.9	−14	0.12	19
Mean	314.7		171.1			85.3
SD	106.9		15.2			66.4
CV	0.34		0.09			0.78

### Compressive strength test

**Fig. 13:**
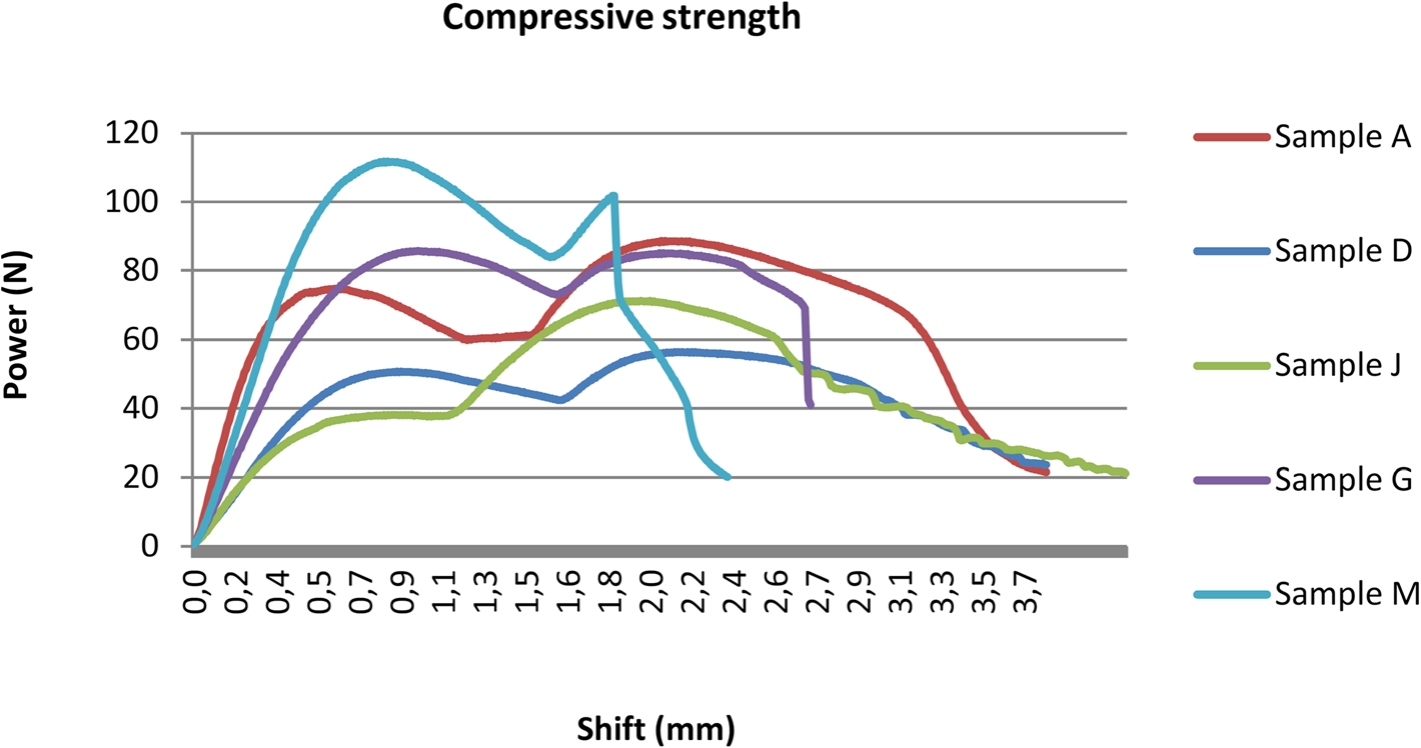
Compressive strength test results

The compressive strength results are shown in Fig. 13 and Table 4. There are two peaks of the curves in case of each samples. The first point is caused by slipping of the samples in the clamp. At the second outstanding point, where the compressive force is at a maximum, the sample snapped. These results illustrate the compressive strength range of the studied polymer.

**Table 4:** Measured compressive forces before and after sterilization

Sterilisation type	Strength (N)	Decreasing (%)
Control	88.6	0
Disinfection	56.4	36
Plasma sterilized	71.9	19
Autoclave 121 °C	85.3	4
Autoclave 134 °C	101.8	−15
Mean	80.8	
SD	17.3	
CV	0.21	

The polymer implant drill guide templates’ measured compressive forces showed low heterogeneity. The Autoclave 134 °C increased the compressive force, but the other sterilisation types decreased it by 4–36%; the worst case was caused by disinfection by 36%.

## Discussion

When placing implants, the most common cause of infection is intraoperative contamination of the open wound. Microorganisms can enter the wound by direct contact or by indirect means, such as microaerosols in the air and incorrectly sterilized instruments [5, 6, 17]. The guidelines for infection control published by the Centers for Disease Control and Prevention (USA) are updated periodically [18]. Medical products can be divided into three major groups depending on the extent to which they may be responsible for the transmission of infection (critical, semi-critical, non-critical). Implant drill guide templates are semi-critical items. The templates may be contaminated with many microorganisms in the dental laboratory during fabrication, and are then used in invasive treatments; therefore, it is crucial that they are sterilized before the implant surgery [5, 6, 19]. If sterilization is inadequate, microorganisms on the drill template can easily enter the area of surgery, causing inflammation and negatively affecting the success of osseointegration and the lifetime of the implant [20]. Therefore, like all other instruments used in implant surgery, drill templates should also be sterilized to avoid infection [5, 6, 18, 19]. According to the guidelines, heat-sensitive semi-critical medical products, such as drill guide templates, require high-level disinfection. High-level disinfection can eliminate many pathogens, but not high levels of bacterial spores [6, 18, 19, 21, 22]. High-level disinfection can be achieved in two ways: using chemical disinfectants or using low-temperature sterilization technology. Chemical disinfectants such as glutaraldehyde, hydrogen peroxide, ortho-phthalaldehyde, and peracetic acid with hydrogen peroxide are suitable for disinfecting, but not sterilizing, heat-sensitive items [6, 19]. Only few publication in the previous 10 years has investigated the sterilization and disinfection of drill guide templates. Sennhenn-Kirchner et al. assessed German practitioners’ treatment of drill guide templates before surgery and evaluated the efficacy of the most commonly used disinfectants. They found that dentists frequently used chlorhexidine and 80% alcohol to disinfect drill guide templates, and that chlorhexidine was not particularly effective [5]. Peter N. Smith et al. estimated the level of microbial contamination found on drill guide templates and tried to evaulated the commonly used antimicrobial potencial of disinfectants. Their study shows that both commercial guides and in-house laboratory guides contained microorganisms prior to disinfection. They found 70% of ethanol for 15 min was effective to eliminate 100% of microorganisms in drill guide templates. The results of Peter N. Smith et al.’s study are similar to those of Sennhenn-Kirchner et al. According to their recommendation the disinfection of surgical guide should be submerged in 70% ethanol for 15 min before surgery [5, 23]. However these studies did not investigate the effect of disinfection on surgical guides. Our results show, as expected, disinfection with Gigasept disinfectant solution did not affect the properties of the material of the tested surgical guide.

In dental offices, the most widely used sterilization method is steam sterilization in an autoclave, in which the sterilization occurs in an enclosed space, at high pressure with the help of saturated steam. There are two types of autoclave settings that are commonly used: normal cycle and fast cycle. Most autoclaves are used according to the following parameters: at a pressure of + 1 bar and a temperature of 121 °C for 20 min or at + 2 bar and 134 °C temperature for 10 min. All heat-resistant and non-corrosive materials may be sterilized in an autoclave [6, 18, 19]. Marei et al. conducted a study to investigate the effect of steam heat sterilization on the dimensional changes of surgical guides. It was concluded that there was no significant influence of steam heat sterilization on the dimensional changes of the tested drill templates [24]. Our results also finds that there was no significant dimensional changes when measured before and after steam sterilization. Futhermore our study investigated two options for sterilizing 3D printed surgical guides and included destructive and non-destructive material tetsing and geometric analysis to investigate the effects of disinfection, plasma sterilization, and autoclave-based steam sterilization on surgical guides. None of the performed examination shows significant effect on the tested surgical guide after steam sterilization at 121 °C.

Shaheen et al. investigate the effect of steam sterilization and hydrogen-peroxid gas plasma sterilization on three 3D printed objects. Tooth replica, surgical cutting guide for the purpose of mandible reconstruction and an orthognatich final splint were printed using PolyJet technology and tested in this study. For each of the three objects, four copies were made: one original STL object, one copy of the object pre-sterilization, one copy after steam sterilization, and one copy after gas plasma sterilization. Each printed object was scanned using a high resolution CBCT protocol. As a result of morphologically and volumetrically evaluation, it was found that morphological changes were noticed with the orthognathic splint object indicating deformation of the printed splints after sterilization. Larger difference was observed with heat sterilization, making it less reliable [25]. Our results is different from the study by Shaheen et al., which showed no significant difference on dimensional changes after steam sterilization. Despite the high temperature in the autoclave, the sterilized specimens did not show any measurable deformation or structural change except hardness of the 134 °C sterilized templates.

Low-temperature sterilization can be used safely for heat-sensitive materials. Sterilization with hydrogen peroxide (H2O2) gas plasma is effective as a germicide, and is also suited for the sterilization of medical devices. Free radicals formed from the hydrogen peroxide can destroy the proteins of microorganisms and pathogens. The sterilization procedure takes place in a vacuum at low temperature (46 ± 4 °C). The advantage of hydrogen peroxide gas plasma sterilization is that the secondary products created during sterilization are nontoxic, and the procedure is safe for the environment. Low-temperature sterilization is used mainly in large hospitals and medical centers, but it is not available or suitable for dental offices [6, 18–20, 26–28]. ISO/NP 22441 (Sterilization of health care products - Low temperature vaporized hydrogen peroxide - Requirements for the development, validation and routine control of a sterilization process for medical devices) standard is under development [29]. Figl et al. also evaulated the deformation of the termolabile splint used computer-navigated surgery undergoing hydrogen peroxide-based low-temperature plasma sterilization. They found that using low-temperature plasma sterilization is an appropiate method for sterilization splints [30]. Our study also showed that low-temperature plasma sterilization does not cause any deformation on the surgical guide. None of the assays show difference between the samples before and after plasma sterilization. Because of the high price and large size of plasma sterilizers, the autoclave is the recommended choice for dental offices. Autoclaves are widely available, even in smaller dental offices, and are less costly than plasma sterilizers.

According to our results both low- and high-temperature sterilization are appropriate methods for sterilizing 3D-printed dental implant drill guide templates. Our study shows that the steam sterilization at 121 °C and plasma sterilization has no significant effects on the dimensional changes and properties of the material of the tested drill template. These findings indicate the necessity of further investigations on the effect of steam sterilization for 3D printed surgical guide. The limitation of this study is that the investigation of the affects of different sterilization and disinfection methods based on only the aspect of material testing. Futher studies are required to be conducted to final conclusions. For future research we would like to enlarge the sample size and include to assess the accuracy of 3D printed surgical guides.

## Conclusions

Within the limitation of our study, it shows that both plasma sterilization and autoclave steam sterilization on 121 °C are suitable for sterilizing the tested 3D printed surgical guides. High temperature steam sterilization did not cause any significant deformation and damages in the tested 3D printed surgical guides.

## Data Availability

The datasets generated and analysed during the current study are available from the corresponding author on reasonable request.

## Abbreviations

3D printing: Three-dimensional printing
ANOVA: Analysis of variance
CV: Coefficient of variation
ETO gas: Ethylene oxide gas
H2O2: Hydrogen peroxide
ISO: International Organization for Standardization
SEM: Scanning electron microscope
Std. dev.: Standard deviation
STL: Stereolithography
